# Effects of rosemary extract supplementation in feed on growth performance, meat quality, serum biochemistry, antioxidant capacity, and immune function of meat ducks

**DOI:** 10.1016/j.psj.2022.102357

**Published:** 2022-11-19

**Authors:** Yuezhou Yao, Yang Liu, Chuang Li, Xuan Huang, Xu Zhang, Ping Deng, Guitao Jiang, Qiuzhong Dai

**Affiliations:** ⁎ Hunan Institute of Animal Husbandry and Veterinary Medicine, Changsha, 410131, China; † Hunan Agricultural University, Changsha, 410125, China

**Keywords:** meat ducks, rosemary extract, serum biochemistry, antioxidant, immune

## Abstract

This study aimed to investigate the effects of diets supplemented with different levels of rosemary extract (**RE**) on the growth performance, meat quality, serum biochemistry, antioxidative capacity, and immunological capacity of Cherry Valley meat ducks. A total of 525 healthy Cherry Valley female meat ducks at 1 d of age were selected for this study. Ducks were randomly divided into 5 treatments with 7 replicates per treatment, and each replicate had 15 ducks. All replicates were randomly assigned to treatments. The study was designed as a dose response experiment. Treatment 1 (**CON**) was fed with the basal diet, and Treatment 2 to 5 (RE250, RE500, RE750, RE1000) were fed with the basal diet supplemented with 250, 500, 750, and 1,000 g/t RE, respectively. The whole experiment lasted 42 days with early stage (1–21 d) and late stage (22–42 d). Results showed that during 22 to 42 d, ducks that were fed over 500 g/t RE had significantly lower feed gain ratio than the ones in CON (*P* = 0.006). In addition, ducks in RE750 had significantly lower L* and a* in leg muscle compared with the ones in CON (*P* < 0.05). Besides, ducks that were fed between 250 and 750 g/t RE had significantly lower total protein level in serum compared with the ones in CON (*P* = 0.005). Ducks in RE250 and RE750 had significantly lower albumin, total cholesterol, high-density lipoprotein cholesterol, and low-density lipoprotein cholesterol levels in serum compared with the ones in CON and RE1000 (*P* < 0.05), and significant quadratic relationships were noticed between albumin, total cholesterol, high-density lipoprotein cholesterol, and low-density lipoprotein cholesterol and dietary RE level (*P* < 0.05). Moreover, ducks that were fed between 500 and 750 g/t RE had significantly higher levels of interleukin-2 in serum compared to the ones in CON and RE1000 (*P* = 0.003). Ducks in RE250 and RE750 had significantly higher levels of immunoglobulin G in serum compared to the ones in CON and RE1000 (*P* < 0.001). Ducks that were fed over 500 g/t RE had significantly higher levels of immunoglobulin A in serum compared to the ones in CON (*P* = 0.001). Finally, ducks that were fed between 500 and 750 g/t RE had significantly higher serum levels of glutathione peroxidase, superoxide dismutase, catalase, and total antioxidant capacity (*P* < 0.05) compared to the ones in CON. Ducks that were fed over 250 g/t RE had significantly lower serum level of malondialdehyde compared to the ones in CON (*P* = 0.020). Collectively, dietary supplementation of RE improved the growth performance and meat qualities of meat ducks during 22 to 42 d, which were possibly associated with the antioxidative and anti-inflammatory effects of RE. Based on the serum antioxidative and immunological parameters, we suggested that 500 to 750 g/t was the optimal supplementation rate for RE in diets for meat ducks aged 22 to 42 d.

## INTRODUCTION

For the past years, the intensive farming system satisfied the increasing demands for poultry products. However, it also caused certain problems to the health of birds, such as physiological stresses and digestive dysfunctions, which reduced the qualities of animal products and jeopardized the benefits of the breeders (Averós and Estevez, 2018). Antibiotic additives had been used in feed to attenuate stresses and promote the growth of birds in the past (Castanon, 2007). But they had led to serious food safety issues, and many countries had banned the utilization of antibiotics in poultry feed (Witte, 2000; Hayes et al., 2004). Therefore, it is now an urge to develop new feed additives replacing antibiotics for the poultry industry.

Rosemary (*Rosmarinus officinalis* L.) is a medicinal plant belonging to the *Lamiaceae* family, whose extract is usually collected from the leaves and flowers by methods of maceration, hydrodistillation, distillation or supercritical fluid extract (Andrade et al., 2018). The phytochemicals in rosemary extract (**RE**) are mainly phenolic compounds, di-, and triterpenes, including rosmarinic acid, camphor, caffeic acid, ursolic acid, carnosic acid, and carnosol (Catherine et al., 2010). Previous papers stated that RE exerted various pharmacological activities such as antibacterial (Bozin et al., 2007), antidiabetic (Bakırel et al., 2008), anti-inflammatory (Yu et al., 2013), antitumor (Tai et al., 2012), and antioxidant effects (PérezFons et al., 2010).

Currently, RE are widely used in alternative medicine and food preservation. Because rosemary is a common, available, and non-toxic herb, RE shows great potential as food and animal feed additives (Oluwatuyi et al., 2004). Previous papers showed inconsistent effects of dietary RE on animals’ growth performances depending on animal species and concentrations of RE supplementations (Turan and Yiğitarslan, 2016; Yildirim et al., 2018). Nonetheless, dietary RE could decrease lipid peroxidation and alleviate oxidative damage in animals, showing a solid antioxidative effect (Yildirim et al., 2018; Yang et al., 2021). The present study was designed to study the dose response of dietary RE on the growth performance, meat quality, serum biochemistry, antioxidative capacity, and immunological capacity of Cherry Valley meat ducks, aiming to provide evidences for the use of RE as meat duck feed additive.

## MATERIALS AND METHODS

The experimental procedures of this study were approved by Hunan Institute of Animal Husbandry and Veterinary Medicine. The RE used in the study is provided by Hunan Shineweare Plant Source Agriculture and Animal Husbandry Co., LTD. (Luxi, Hunan, China), which was obtained from rosemary leaves using solvent extraction method. Briefly, rosemary leaves were dried in the shade for 4 d, and powdered in a blender. Every 100 g of plant sample was extracted with 500 mL ethanol at room temperature. The extract was filtered, evaporated, and dried in a vacuum at 40°C with a rotary evaporator after 48 h, then the organic solvents were evaporated under reduced pressure and temperature. The dried extracts were stored at 4°C until use.

### Experimental Design and Diets

A total of 525 healthy Cherry Valley female meat ducks at 1 d of age were selected for this study. The ducks were housed on netting cages with the dimension of 1.8 m × 1.2 m × 2 m (15 ducks per cage), free accessed to water and feed. Artificial illumination was used and the experimental facility was under regular sanitation during the entire experimental period. Ducks were randomly divided into five treatments with seven replicates per treatment, and each replicate had 15 ducks. Each cage was treated as a replicate. Treatment 1 (CON) was fed with the basal diet, and Treatment 2 to 5 (RE250, RE500, RE750, RE1000) were fed with the basal diet supplemented with 250, 500, 750, and 1,000 g/t RE, respectively. All dietary ingredients were milled to powders, then thoroughly mixed in a mixing machine, and finally pelleted to granules with 2.5 mm particle size. The whole experiment lasted 42 d with early stage (1–21 d) and late stage (22–42 d). The composition and nutrient contents of the basal diets at different stages were shown in Table 1.

**Table 1:** Composition and nutrient contents of basal diets^1^ (dry matter basis, %).

Items	Diets	
	Early stage (1–21 d)	Late stage (22–42 d)
Ingredients		
Corns	43.20	47.00
Flour	10.00	10.00
Soybean meal	28.00	20.00
Wheat middlings	10.00	10.00
Corn gluten meal (CP 60%)	3.00	3.00
Soybean oil	1.00	5.50
Limestone	1.50	1.50
CaHPO_4_	1.30	1.00
NaCl	0.30	0.30
70% L-Lysine H_2_SO_4_	0.83	0.86
98% DL-Methionine	0.23	0.20
98% L-Threonine	0.22	0.22
Vitamin complex	0.03	0.03
Trace element complex	0.10	0.10
50 % Choline chloride	0.10	0.10
Enzyme complex	0.02	0.02
Mildewproof agent	0.10	0.10
Antioxidant	0.02	0.02
High temperature resistant phytase (10^4^ IU/g)	0.02	0.02
Probiotics (10^10^ counts/g)	0.02	0.02
Glucose oxidase (10^4^ IU/g)	0.02	0.02
Total	100.00	100.00
Nutrient contents		
Metabolic energy, MJ/kg	12.05	13.39
Crude protein	20.52	17.36
Calcium	1.00	0.90
Total phosphorus	0.62	0.53
Available phosphorus	0.36	0.29
Crude fiber	5.18	4.77
Ether Extract	3.47	7.86
Lysine	1.36	1.18
Methionine	0.50	0.44
Threonine	0.91	0.79
Tryptophan	0.21	0.17

a Nutrient contents are calculated values.

### Growth Performance

On d 1, 21, and 42, body weight (**BW**) of each experimental duck was measured after fasting for 12 h. Feed intake and leftover per cage were recorded daily. The average daily weight gain (**ADG**), average daily feed intake (**ADFI**), and feed gain ratio (**F/G**) were calculated by cages.

### Serum Biochemical Parameters

On d 42, one duck from each cage (seven ducks per treatment) with body weight close to the mean was chosen for blood collection. Blood samples were obtained from wing veins by sterilized syringes and kept in separate tubes. After maintained at room temperature for 1 h, all blood samples were centrifugated at 4,000 × *g* for 5 min to separate the serum, which were stored at −20°C for further analysis.

The levels of glucose (**GLU**), total protein (**TP**), albumin (**ALB**), urea nitrogen (**BUN**), uric acid (**UA**), triglyceride (**TG**), total cholesterol (**TCHO**), high density lipoprotein cholesterol (**HDL-C**), low density lipoprotein cholesterol (**LDL-C**), and free fatty acid (**FFA**) in serum were measured with the commercial assay kits (Hangzhou Adicon Medical Laboratory Center Co., LTD, Hangzhou, China) and an automatic biochemical analyzer (URIT-8000, Hangzhou Adicon Medical Laboratory Center Co., LTD, Hangzhou, China).

### Serum Immunological and Antioxidative Parameters

The serum levels of immunological parameters included concentrations of interleukin-2 (**IL-2**), immunoglobulin A (**IgA**), immunoglobulin G (**IgG**), and immunoglobulin M (**IgM**), and antioxidative parameters included concentrations of glutathione peroxidase (**GSH-Px**), superoxide dismutase (**SOD**), catalase (**CAT**), malondialdehyde (**MDA**), and total antioxidant capacity (**T-AOC**). These parameters were determined by the commercial assay kits (Nanjing Jiancheng Bioengineering Institute, Nanjing, China) and an automated fluorescence instrument (Multiskan Skyhigh, Thermo Fisher Scientific, Waltham, MA).

### Meat Quality

After collecting blood, the sampled ducks were then immediately slaughtered by cervical dislocation. The carcasses were de-feathered manually, then 2-cm-thick samples of right-side leg muscle and breast muscle were cut from each duck for meat quality analysis.

The meat colors were determined by a colorimeter (CR-400, Minolta Camera Co., Osaka, Japan) as L*, a*, and b* after 45-min postmortem, which were indicators of lightness, red/green and yellow/blue, respectively.

The water-holding capacity, cooking loss, and shear force of meat samples at 45-min postmortem were assessed according to the method described previously (Qiao et al., 2017). Briefly, the water-holding capacity of meat samples were represented by the drip loss. Meat samples were cut into slices of about 1 cm thick, with a diameter of 2.5 cm, wrapped in a layer of gauze, placed between filter papers and exposed to a force of 35 kg for 5 min by a determinator (MAEC-18, Nanjing Mingao Instrument Co., Nanjing, China). The drip loss was calculated as the difference in sample weights before and after squeezing, divided by the sample weight before squeezing.

To determine the cooking loss, meat samples were cut at size of about 3 × 3 cm, weighted and placed in a boiling bag in a water bath at the temperature of 80°C, and were removed from bath when the internal temperature reached 75°C. After cooling to room temperature in cold water, the samples were patted dry with filter paper and re-weighted. Cooking loss were calculated as the difference in sample weights before and after cooking, divided by the sample weight before cooking.

After the determination of cooking loss, the cooked meat samples were cut into strips at size of 3 × 1 × 1 cm, parallel to the direction of the muscle fibers and were sheared perpendicularly to the longitudinal of the myofibers using a Texture Analyzer (C-LM4-1, Beijing Bulader Tech Co., Beijing, China).

### Statistical Analysis

Cage was used as the experimental unit. Statistical analysis of data were done with Statistical Package for the Social Sciences (SPSS) 19.0 (IBM, Armonk). One-way ANOVA followed by Duncan's multiple range test were performed to test the significant mean differences among treatments. Polynomial orthogonal contrasts were applied to determine linear and quadratic responses of measured parameters to the dietary RE concentration. All results were presented as means and pooled standard errors of the means (**SEM**). A probability of *P* < 0.05 was considered significant, and 0.05 < *P* < 0.1 was considered having trend.

## RESULTS

### Growth Performance

The effects of dietary RE supplementation on the BW, ADG, ADFI, and F/G of meat ducks are shown in Table 2. No mortality was observed during the whole experiment period. During 1 to 42 d, the ADG, ADFI and F/G of ducks were not affected by RE supplementation (*P* > 0.05). However, during 22 to 42 d, ducks that were fed over 500 g/t RE had significantly lower F/G than the ones in CON (*P* = 0.006), and significant linear (*P* = 0.001) and quadratic relationships (*P* = 0.001) were noticed between F/G and dietary RE level. Besides, dietary RE showed trends in increasing the ADFI (*P* = 0.061) and F/G (*P* = 0.051) of meat ducks during 1 to 21 d, and increasing the ADG of meat ducks during 22 to 42 d (*P* = 0.090). These data revealed that dietary supplementation of RE could improve the feed efficiency for meat ducks during 22 to 42 d.

**Table 2:** Effect of dietary RE on the growth performance of meat ducks.

Item	Treatment					SEM	*P* value		
	CON	RE250	RE500	RE750	RE1000		ANOVA	Linear	Quadratic
Initial BW (1 d), g	51.798	51.963	51.827	51.637	52.020	0.052	0.139	0.775	0.634
Final BW (21 d), g	1024.507	1029.524	1034.184	1014.566	1039.727	3.779	0.264	0.583	0.753
Final BW (42 d), g	2938.572	2980.953	2985.169	3004.511	3016.939	9.755	0.126	0.009	0.030
1–21 d									
ADG, g	46.318	46.551	43.781	45.854	47.031	0.180	0.281	0.588	0.761
ADFI, g	66.198	68.547	68.763	69.130	70.053	0.421	0.061	0.006	0.016
F/G	1.422	1.453	1.471	1.506	1.491	0.010	0.051	0.004	0.011
22 - 42 d									
ADG, g	91.147	92.924	92.904	94.759	94.154	0.434	0.090	0.011	0.029
ADFI, g	188.630	186.566	185.687	184.009	184.473	1.091	0.732	0.176	0.365
F/G	2.070^a^	2.010^ab^	1.999^b^	1.943^b^	1.960^b^	0.012	0.006	0.001	0.001
1 - 42 d									
ADG, g	68.733	69.739	69.840	70.307	70.591	0.232	0.127	0.009	0.031
ADFI, g	127.788	127.920	127.584	127.666	127.976	0.678	1.000	0.979	0.991
F/G	1.860	1.836	1.826	1.816	1.813	0.008	0.371	0.046	0.112

a,b Mean values within a row bearing different superscripts differ significantly (*P* < 0.05). Abbreviations: ADG, average daily weight gain; ADFI, average daily feed intake; BW, body weight; F/G, feed gain ratio.

### Meat Quality

The effects of dietary RE supplementation on the parameters representing meat quality of the meat ducks are shown in Table 3. On 42 d, the meat qualities of breast muscle were not affected by dietary RE (*P* > 0.05). However, ducks in RE750 had significantly lower L* in leg muscle compared with the ones in CON (*P* = 0.032), and significant quadratic relationship was noticed between L* and dietary RE level (*P* = 0.036). Besides, ducks that were fed between 500 and 750 g/t RE had significantly higher a* in leg muscle compared to the ones in RE1000 (*P* = 0.029), and significant quadratic relationship was noticed between a* and dietary RE level (*P* = 0.032). In addition, dietary RE showed a trend in influencing the water-holding capacity in leg muscle of meat ducks (*P* = 0.051). These data suggested that dietary RE could influence the color of leg muscle in meat ducks.

**Table 3:** Effect of dietary RE on the meat quality of meat ducks.

		Treatment					SEM	*P* value		
Item		CON	RE250	RE500	RE750	RE1000		ANOVA	Linear	Quadratic
Breast muscle										
Meat color	L*	36.885	36.493	36.421	37.542	37.015	0.476	0.954	0.271	0.546
	a*	10.549	10.252	11.386	10.214	11.435	0.263	0.383	0.545	0.806
	b*	4.190	3.577	4.591	3.970	4.664	0.203	0.445	0.349	0.594
Water-holding capacity		42.977	44.299	41.019	44.592	42.614	0.550	0.233	0.905	0.992
Cooking loss, %		27.172	25.209	26.853	27.637	23.373	0.775	0.404	0.391	0.404
Shear force, N		32.498	34.330	32.209	35.443	34.082	0.581	0.386	0.356	0.610
Leg muscle										
Meat color	L*	41.662^a^	40.160^ab^	39.141^ab^	37.630^b^	40.208^ab^	0.419	0.032	0.150	0.036
	a*	9.069^ab^	9.157^ab^	10.721^a^	10.130^a^	8.266^b^	0.276	0.029	0.530	0.032
	b*	5.474	5.473	6.999	6.151	5.595	0.251	0.243	0.520	0.128
Water-holding capacity		33.027	30.964	35.188	29.922	33.555	0.637	0.051	0.646	0.900
Cooking loss, %		15.500	16.414	17.985	18.292	18.105	0.904	0.857	0.278	0.486
Shear force, N		18.253	21.331	21.980	22.251	20.167	0.532	0.157	0.357	0.036

a,b Mean values within a row bearing different superscripts differ significantly (*P* < 0.05).

### Serum Biochemical Parameters

The effects of dietary RE supplementation on the serum biochemical parameters of the meat ducks are shown in Table 4. On 42 d, ducks that fed between 250 to 750 g/t RE had significantly lower TP level in serum compared with the ones in CON (*P* = 0.005), and significant quadratic relationship was noticed between TP and dietary RE level (*P* = 0.009). Besides, ducks in RE250 and RE750 had significantly lower ALB level in serum compared with the ones in CON and RE1000 (*P* = 0.038), and significant quadratic relationship was noticed between ALB and dietary RE level (*P* = 0.024). Moreover, ducks in RE250 and RE750 had significantly lower TCHO (*P* = 0.001), HDL-C (*P* = 0.003), and LDL-C (*P* = 0.001) levels compared with the ones in CON and RE1000, and significant quadratic relationships were noticed between TCHO (*P* = 0.005), HDL-C (*P* = 0.002), and LDL-C (*P* = 0.012) and dietary RE level. These data suggested that moderate level of dietary RE could influence the serum biochemical indexes, especially the protein and lipid metabolism in meat ducks.

**Table 4:** Effect of dietary RE on the serum biochemical parameters of meat ducks.

Item	Treatment					SEM	*P* value		
	CON	RE250	RE500	RE750	RE1000		ANOVA	Linear	Quadratic
GLU, mmol/L	5.370	4.677	6.219	5.236	6.003	0.253	0.305	0.304	0.588
TP, g/L	31.933^a^	23.014^c^	26.271^bc^	24.686^bc^	28.500^ab^	0.842	0.005	0.523	0.009
ALB, g/L	12.450^a^	9.414^b^	10.533^ab^	9.514^b^	12.043^a^	0.410	0.038	0.929	0.024
BUN, mmol/L	0.642	0.610	0.576	0.681	0.637	0.013	0.113	0.467	0.472
UA, μmol/L	225.333	232.571	242.857	205.857	251.429	10.624	0.720	0.748	0.898
TG, mmol/L	0.640	0.559	0.626	0.629	0.623	0.029	0.922	0.830	0.929
TCHO, mmol/L	3.423^a^	2.134^b^	2.819^ab^	2.379^b^	3.393^a^	0.134	0.001	0.698	0.005
HDL-C, mmol/L	1.927^a^	1.324^b^	1.547^b^	1.446^b^	1.933^a^	0.068	0.003	0.638	0.002
LDL-C,mmol/L	1.743^a^	1.023^c^	1.427^ab^	1.116^bc^	1.647^a^	0.073	0.001	0.994	0.012
FFA, μmol/L	1094.377	1069.226	837.626	735.051	940.673	57.109	0.231	0.125	0.137

a-c Mean values within a row bearing different superscripts differ significantly (*P* < 0.05). Abbreviations: ALB, albumin; BUN, urea nitrogen; FFA, and free fatty acid; GLU, glucose; HDL-C, high density lipoprotein cholesterol; LDL-C, low density lipoprotein cholesterol; TCHO, total cholesterol; TG, triglyceride; TP, total protein; UA, uric acid.

### Serum Immunological and Oxidative Stress-related Parameters

The effects of dietary RE supplementation on the serum immunological and oxidative stress-related parameters of the meat ducks are shown in Table 5. Regarding to the immunological parameters, ducks that were fed between 500 and 750 g/t RE had significantly higher IL-2 levels in serum compared to the ones in CON and RE1000 (*P* = 0.003). Besides, ducks in RE250 and RE750 had significantly higher IgG levels in serum compared to the ones in CON and RE1000 (*P* < 0.001). In addition, ducks that were fed over 500 g/t RE had significantly higher IgA levels in serum compared to the ones in CON (*P* = 0.001). Moreover, significant quadratic relationships were noticed between IL-2 (*P* = 0.001), IgG (*P* < 0.001), and IgA (*P* = 0.004) levels and dietary RE level, significant linear relationship was noticed between IgA levels and dietary RE level (*P* = 0.017).

**Table 5:** Effect of dietary RE on the serum immunological and oxidative stress-related parameters of meat ducks.

Item	Treatment					SEM	*P* value		
	CON	RE250	RE500	RE750	RE1000		ANOVA	Linear	Quadratic
IL-2, pg/g	31.683^b^	37.645^ab^	43.001^a^	44.055^a^	34.474^b^	1.284	0.003	0.251	0.001
IgM, μg/g	107.789	107.640	110.332	98.135	107.749	2.845	0.713	0.641	0.880
IgG, μg/g	354.389^c^	454.673^a^	431.207^ab^	456.456^a^	383.764^bc^	9.830	<0.001	0.532	<0.001
IgA, μg/g	40.273^c^	46.728^bc^	55.672^a^	47.720^b^	51.212^ab^	1.299	0.001	0.017	0.004
GSH-Px, ng/g	153.501^c^	177.961abc	200.752^a^	194.186^ab^	169.012^bc^	5.119	0.020	0.257	0.003
MDA, nmol/g	1.703^a^	1.193^b^	1.188^b^	1.118^b^	1.326^b^	0.053	0.002	0.044	<0.001
SOD, ng/g	1.268^c^	1.501^ab^	1.701^a^	1.691^a^	1.387^bc^	0.041	<0.001	0.207	<0.001
CAT, pg/g	2.125^c^	2.593^bc^	2.720^ab^	3.192^a^	2.383^bc^	0.094	0.002	0.128	0.003
T-AOC, U/mL	31.767^c^	37.520^bc^	47.401^a^	42.890^ab^	36.452^bc^	1.427	0.002	0.198	0.001

a-c Mean values within a row bearing different superscripts differ significantly (*P* < 0.05). Abbreviations: CAT, catalase; GSH-Px, glutathione peroxidase; IL-2, interleukin-2; IgA, immunoglobulin A; IgG, immunoglobulin G; IgM, immunoglobulin M; MDA, malondialdehyde; SOD, superoxide dismutase; T-AOC, total antioxidant capacity.

Regarding to the oxidative stress-related parameters, ducks that were fed between 500 and 750 g/t RE had significantly higher antioxidative enzymes such as GSH-Px (*P* = 0.020), SOD (*P* < 0.001), and CAT (*P* = 0.020) levels, and antioxidant capacity such as T-AOC (*P* = 0.020) level compared to the ones in CON. Besides, ducks that were fed over 250 g/t RE had significantly lower oxidative damage such as MDA level compared to the ones in CON (*P* = 0.020). Moreover, significantly quadratic relationships were noticed between the measured oxidative stress-related parameters and dietary RE level (*P* < 0.01), and significant linear relationships was noticed between MDA level in serum and dietary RE level (*P* = 0.044).

## DISCUSSION

With the restriction of antibiotics in poultry feeds, researchers were trying to explore new feed additives to promote the poultry growth and create more benefits for the breeders. Plant extracts with multiple bioactive phytochemicals have shown great potential as feed additives (Valenzuela-Grijalva et al., 2017). Rosemary as a medicinal plant has been widely used in traditional medicine, fragrance and food industries (Viuda-Martos et al., 2010). Its extract is mainly composed of flavonoids, di, and triterpenoids, monoterpenes, sesquiterpenes, alcohol, ester, ketone, hydroxycinnamic derivatives and others (Ribeiro-Santos et al., 2015). Furthermore, rosemary extract was safe to be used as food additives for human beings and animals, that it was authorized in food with established maximum levels in European Union. Ghazalah and Ali (2008) reported that diet contains 1% and 2% ground rosemary leaves increased the weight gain and feed intake, and reduced the feed to gain ratio of broilers from 28 to 49 d. Yesilbag et al. (2011) fed broilers with diets supplemented with rosemary volatile oil yielded by steam distillation method, and found that the addition of rosemary volatile oil did not impair the growth performances of broilers during the experiment trials (0 to 21 d, 21 to 42 d, and 0 to 42 d), but increased the live weight gain and decreased the feed conversion rate of broilers during 0 to 21 d. The present study showed similar results as the previous, that dietary RE showed linear and quadratic trends in improving the ADG of meat ducks from 1 to 21 d (*P* = 0.061), and 22 to 42 d (*P* = 0.090). Moreover, dietary RE significantly reduced the F/G of meat ducks from 22 to 42 d (*P* = 0.006). These studies suggested that rosemary and its products (volatile oil and extracts) had potential in promoting the growth of poultry as feed additives. However, Yildirim et al. (2018) had inconsistent result that administration of 100 and 200 mg/kg diet rosemary ethanol extract reduced the body weight and feed consumption of broilers from 1 to 42 d. It might be caused by the differentiations in rosemary processing methods. The qualitative and quantitative studies on bioactive compounds isolated from rosemary depend greatly on proper choice of processing. Factors including plant portions, plant conditions, extraction methods, equipment settings, and actives doses would eventually affect the experimental results (Azmir et al., 2013). It was possible that the ethanol extract of rosemary did not receive enough bioactive phytochemicals than the other methods to implement the growth promotional effects.

Duck meat had relatively higher fat and muscle fiber, and lower protein contents compared to chicken (Jaturasitha et al., 2008). It was also a good source of polyunsaturated fatty acids (Ali et al., 2007). Driven by the demand of processed foods by consumers, the global duck meat market was growing at a steady pace, which reached a value of about $ 11.23 billion in 2020 (Biswas et al., 2019). Meat qualities determined the economic and nutritional value of the meat products. In the current study, dietary RE quadratically decreased the lightness (L*) (*P* = 0.032) and increased the redness (a*) (*P* = 0.029) of the leg muscle in meat ducks. Variations of meat color were dominated by the myoglobin, which changes color depending on its biochemical state, especially the degree of oxidation or reduction of the myoglobin (Suman and Joseph, 2013). Decreasing lightness value and increasing redness were descriptors of meat color improvement, which were reported to be related to decreasing in metmyoglobin formation (Nieto et al., 2010). It hypothesized from the result of the current study that dietary RE at 750 g/t reduced the lipid oxidation and postponed the deterioration of the leg muscle. Similar results were obtained in a study incorporating rosemary essential oil to lambs (Smeti et al., 2018). Another study in broilers fed with smashed dry rosemary leaves and rosemary volatile oil showed no differences in broiler meat colors among control and rosemary-treated groups (Yesilbag et al., 2011), possibly because that the different methods of processing raw materials led to variations in the antioxidative capacities of rosemary products.

Biochemical parameters in serum were indicators of the internal environment of the birds. TP and ABL levels were indicators for hepatic function (Chen et al., 2014). RE was reported to have effects in reducing toxicity and regulating lipid metabolism in the liver (Almakhatreh et al., 2019; Wang et al., 2019). Ghazalah and Ali (2008) found that 5% rosemary leaves supplementation in feed increased the TP and ALB levels in blood of broiler chicken. Yildirim et al. (2018) also found that feed supplemented with 100 and 200 mg/kg rosemary ethanol extracts increased the TP and ALB levels in serum of broiler chicken. However, the present study showed contradictory result that feed supplemented with 250 and 750 g/t RE reduced the serum TP and ALB levels in meat ducks. It was possible that the differences in digestion habits as well as the physiological traits of ducks and chicken led to inconsistent results in effects of hepatic function or TP digestibility. Besides, academic publications stated that different rosemary forms (powder, leaf, extract and oil) possessed different concentrations of active ingredients, which might arise contrary results in the studies (Andrade et al., 2018). On the other hand, feed supplemented with RE quadratically reduced the levels of TCHO, HDLC-C, and LDL-C in serum of meat ducks, that 250 and 750 g/t dietary RE showed significant effects in reducing such parameters compared with the control in the present study (*P* = 0.001, 0.003, 0.001, respectively). It suggested a strong regulation effect of dietary RE in meat ducks’ lipid metabolism as previously reported (Hassani et al., 2016). Similar results were published in a study in Zucker rats that 0.5% RE supplemented-diet reduced levels of TC, LDL-C, and HDL-C in rats (Romo Vaquero et al., 2012). Rosemary herbs and essential oils also showed regulation effects on lipid metabolism in laying hens (Alagawany and Abd El-Hack, 2015; Torki et al., 2018). The possible mechanism of the hypolipidemic effects of RE was that RE could regulate PPAR-γ by activating the signaling pathways including AMP-activated protein kinase (**AMPK**) and PPAR-γ, and upregulating the LDL-C receptor, sirtuin 1 (increases fatty acid oxidation), and PGC1α (activates PPAR-γ) (Zheng et al., 2013). Further study regarding to carnosic acid (**CA**), which was one of the main active ingredients in RE, implied that CA also increased the PPAR-γ mRNA expression and suppressed the activation of nuclear factor-κB (**NF-κB**), that contributed to the improvement of lipid metabolism (Tsai et al., 2014).

Besides biochemical parameters, antioxidative and immunological cytokines were also important indicators reflecting the health status of birds. The immunological and oxidative stresses were inevitable in birds raised in intensive farming system, that they disturbed the redox balance, triggered the innate and adaptive immune responses, and eventually took up excessive nutrition and energy from growth (Lauridsen, 2019). Within the organism, an antioxidant defense system regulated the redox balance, which was consisted of enzymatic components such as SOD, CAT and GSH-Px, and non-enzymatic components such as GSH (Surai et al., 2019). On the other hand, Lymphocyte cells in avian innate immune system were able to produce a diversity of antibodies, such as IgM, IgG, and IgA, to serve as the first line to fight against the inflammation (Berghof et al., 2018). In adaptive immune system, type-1 helper T-cells (Th1) bound to the macrophage and released IL-2, which activated the bounded T-cells to eliminate inflammation (Jacob and Pescatore, 2017). Therefore, levels of such protective cytokines were associated with birds’ antioxidative and anti-inflammatory capabilities. In the present study, dietary RE quadratically increased the levels of IL-2 (*P* = 0.003), IgG (*P* < 0.001), IgA (*P* = 0.001), GSH-Px (*P* = 0.020), SOD (*P* < 0.001), CAT (*P* = 0.002), and T-AOC (*P* = 0.002) in serum of meat ducks, and the supplemented rate of RE between 500 and 750 g/t significantly increased the levels of above cytokines compared to the control which suggested that dietary RE between 500 and 750 g/t could improve the antioxidative and anti-inflammatory capacities of meat ducks. Moreover, compared with the ones in control, dietary RE treated meat ducks had significantly lower serum level of MDA, which was a reactive aldehyde formed by ROS degrading the polyunsaturated lipids in the organism, and considered as a common index of oxidative damage in animal (Jing et al., 2013). These data proved that RE had effects of improving antioxidant system, alleviating oxidative damage, and balancing redox homeostasis of serum in meat ducks. Previous studies had proved such effects of RE in pigs (Liotta et al., 2015), quails (Cetin et al., 2017), and food preservation (Sebranek et al., 2005). Moreover, an anti-inflammatory effect of RE had also been found in rats (Silva et al., 2015) and in-vitro model (Yi and Wetzstein, 2010). The active components in RE mainly contributed to functions of oxidative damage alleviation and antioxidant enzyme enhancement, including classes of phenolic acids, flavonoids, diterpenoids and so on (Nieto et al., 2018). The isoprenoid quinones in components of RE could act as chain terminators of free radicals and chelators of ROS (Wu et al., 1982), and the phenolic compounds turned lipid and hydroxyl radicals into stable products (Gordon, 1990). Possible mechanisms of the anti-inflammatory effect of RE were concluded as inhibition of cyclooxygenases enzyme (COX-2), inhibition of NO production, and decline of TNF-α (Kuo et al., 2011).

## CONCLUSION

In conclusion, dietary supplementation of RE improved the growth performance of meat ducks at 1 to 21 d, and enhanced the meat qualities of leg muscle, which were possibly associated with the antioxidative and anti-inflammatory effects of RE. Quantity effects of dietary supplemented RE were obvious, as significant quadratic relationships were noticed between the measured parameters and dietary RE levels. Based on the serum antioxidative and immunological parameters, we suggested that 500 to 750 g/t was the optimal supplementation rate for RE in diets for meat ducks aged 1 to 42 d.
